# I Get Knocked Down but I Get Up Again: Autonomous Motivation Sustains Identification and Collective Action After (Specific) Failure

**DOI:** 10.1177/01461672251381184

**Published:** 2025-11-03

**Authors:** Lisette Yip, Emma F. Thomas, Catherine Amiot, Léïla Eisner, Morgana Lizzio-Wilson, Winnifred R. Louis, Craig McGarty, Fathali Moghaddam

**Affiliations:** 1 Flinders University, Bedford Park, South Australia, Australia; 2 Université du Quebec à Montreal, Quebec, Canada; 3 University of Zurich, Switzerland; 4 University of Exeter, UK; 5 University of Queensland, St. Lucia, Australia; 6 Western Sydney University, New South Wales, Australia; 7 Georgetown University, Washington, DC, USA

**Keywords:** collective action, self-determination theory, social identity, social movement failure

## Abstract

Movements often experience setbacks while striving to achieve (or prevent) social change. We examined whether autonomous motivation—which captures supporters’ internalized commitment to a cause—would sustain identification with the movement and collective action after experiencing failure (vs. success) outcomes following the marriage equality vote in Australia (Study 1; *N* = 186), and an experimental induction of movement failure (Study 2; *N* = 137). Autonomous motivation positively predicted identification and collective action, but there was no evidence of moderation by outcome. In Study 3 (*N* = 377), we experimentally manipulated outcomes (success/failure) and framing (specific/broad) of the climate action movement. We found evidence of a three-way interaction such that the effects of autonomous motivation on identification were strongest after a specific campaign failure. We conclude that autonomous motivation can help to buffer the demotivating effects of a specific failure as well as sustaining identification and commitment to action broadly.

Movements seeking to achieve (or prevent) dramatic social change often face obstacles as their supporters strive to achieve their desired goals. Failure can be experienced as demotivating, and for some supporters, it can lead to disengagement with the movement (Lizzio-Wilson et al., 2021; Louis et al., 2022). To achieve meaningful social change, supporters must continue investing effort and pursuing the movement’s goals, even after experiencing setbacks (Selvanathan & Jetten, 2020). In the current research, we seek to understand when people who support social movements “push through” failure and remain committed to supporting the cause. We examine the quality of underlying motivation as a factor which may help us understand when people are more likely to continue engaging in collective action after failure. Specifically, we draw on the joint insights of self-determination theory (Deci & Ryan, 1985; Ryan & Deci, 2000) and the social identity approach (Tajfel & Turner, 1979; Turner et al., 1987) to argue that people will persist in collective action, even in the face of failure, when they value the movement’s goals and hold them to be personally important—that is, when they experience their involvement as autonomously motivated. We test these claims using a quasi-experiment examining supporters of movements for and against marriage equality in Australia (Study 1), an experimental test involving a sample of supporters of various social causes in Australia (Study 2), and an experimental test focused on combating climate change in the United States (Study 3).

## Autonomous Motivation Sustains Collective Action After Failure

Self-determination theory (Deci & Ryan, 1985) posits that individuals’ motivation for engaging in a behavior can vary in the extent to which it is self-determined and performed of one’s own volition. On one hand, behavior can be *autonomously motivated*, meaning that it is personally important and stems from the individual’s goals, values, and sense of self (Ryan & Deci, 2000). In the context of collective action, an autonomously motivated supporter may engage in behaviors to support a cause because they are deeply invested in better outcomes for marginalized groups and feel that the movement’s goals align with their personal values. For example, someone might identify as a supporter of rights for LGBTQI+ people because this movement aligns with their personal values that all individuals have inherent worth and the right to equality, justice, and dignity. In contrast, behavior can be driven by *controlled motivation*, when it is performed to attain rewards or punishments or influenced by external forces, and is not representative of the individual’s internalized desires. For example, someone driven by controlled motivation may take action to promote LGBTQI+ rights to alleviate their own feelings of guilt or to appear prosocial and be liked by others.

Relative to controlled motivation, autonomous motivation is associated with greater persistence over time in a variety of contexts. Autonomous motivation predicts ongoing participation in sport (Hutmacher et al., 2020), health care behaviors (Tam et al., 2019), and pursuit of academic goals (Holding et al., 2024). Although there is a rich literature addressing the role of autonomous motivation in sports, health, education, and the workplace, the literature in relation to political behaviors is relatively nascent. However, one study showed that autonomous motivation predicted ongoing participation in collective action to combat global poverty over time (Yip et al., 2024). Thus, autonomous motivation appears to be important for sustaining behavior.

Other research more directly addresses the relationship between autonomous motivation and persistence after setbacks. In a study by Holding et al. (2017), students who initially reported higher levels of autonomous motivation were more willing to exert effort, less likely to doubt whether they should continue pursuing goals after setbacks, and were ultimately more likely to experience continued goal pursuit. Autonomous motivation is also associated with lower perceptions that obstacles are problematic to goal pursuit, compared to controlled motivation (Leduc-Cummings et al., 2022; Milyavskaya et al., 2015). Because autonomous goals are more valued and salient than controlled goals, they are more resistant to the temptation to disengage in the face of difficulty (Sheldon & Elliot, 1998).

In the current research, we consider the context of collective social and political goals, a relatively novel domain in the literature on self-determination theory (see Ryan & Deci, 2017). Collective action is defined as any action that is taken with the aim of advancing a group’s goals (Wright et al., 1990). In this paper, we focus specifically on conventional collective actions, that is, those that are legal, common, and approved of by authorities (Louis et al., 2020). We expect that autonomous motivation will be associated with persistence in collective action after failure; that is, it will protect against the demotivating effects of failure. In contrast, autonomous motivation may not be necessary to sustain commitment after success, as success has a positive, recursive influence on efficacy and future action (Bilali et al., 2025; Burrows et al., 2023). We also consider the impacts of autonomous motivation on group-based processes relevant to collective action, specifically the social identification that drives commitment to group goals (Thomas et al., 2022).

## Does Autonomous Motivation Sustain Opinion-Based Group Identification After Failure?

### The Role of Identification

Social movements scholarship emphasizes the importance of collective identities as drivers of social movement participation (Flesher Fominaya, 2010; Polletta & Jasper, 2001) and retention of supporters over time and through challenges. Collective identities foster loyalty not only to the cause, but to the group, its members, and its leaders, and thus can drive continued action even when the costs are high and rewards are minimal (Bunnage, 2014). In the present research, we draw on social identity theory’s conceptualization of opinion-based groups, where group membership is based on shared opinions about how the world should be (e.g., supporters of climate action, pro-marriage equality, anti-marriage equality; see Bliuc et al., 2007, 2015). As such, opinion-based groups can include members of both structurally advantaged and disadvantaged groups who share an opinion about a desired change, and are a key driver of collective action (e.g., Lizzio-Wilson et al., 2025).

Social identity theory suggests that people define themselves in terms of their group memberships and view these groups as part of who they are; they are therefore motivated to take action to enhance the success of the group (Tajfel & Turner, 1979). Stronger identification with a social group is predictive of greater engagement in collective action (e.g., Simon et al., 1998; van Zomeren et al., 2008) and continued identification with groups even when they are not successful (Ellemers et al., 1997; Spears et al., 1997). Thus, social identification is key to sustaining group-based action. Yet, to understand when collective action is sustained through failure, it is also necessary to understand what drives the strength of identification.

To consider supporters’ motivation for engaging in collective action, it is important to consider how behavior is internalized within the social-level self, and how *collective* (rather than individual-level) goals are experienced as internalized and personally important (Amiot et al., 2020; Kachanoff et al., 2019; Thomas et al., 2019; Yip et al., 2024, 2025). We integrate insights from self-determination theory and social identity theory as a basis to consider how individuals are motivated to participate in group-based action.

### Autonomous Motivation Facilitates Opinion-Based Group Identification

We propose that autonomous motivation will play a key role in sustaining opinion-based group identification, and therefore collective action, after experiencing failure. Autonomous motivation and opinion-based group identification are interlinked, but distinct constructs. The literature on social identification tends to focus on the *strength* of identification, such that people can vary in the degree to which they see themselves as part of the group (Tajfel & Turner, 1979). However, incorporating the insights of self-determination theory suggests that people may also have a different motivational *quality* to their support for social movements and, in the context of opinion-based group identities, this may drive the strength of identification. Those who experience their support for a cause as integral to their sense of self and personal values (i.e., autonomous rather than controlled) would be expected to identify more strongly with a social group whose identity is derived from shared support for the cause. As such, autonomous motivation and opinion-based group identification are interlinked and significantly associated, but nevertheless conceptually distinct (see Yip et al., 2024).

We therefore expect that when the group goals are internalized and personally important (reflecting a high degree of autonomous motivation), supporters will be more likely to strengthen identification with their group after experiencing a setback, as they are highly committed to the group identity (e.g., Blackwood & Louis, 2012; Ellemers et al., 1997). However, when the group experiences success, the degree of internalized motivation may be less important in sustaining opinion-based group identification as there is no threat to this identity, and no motive to disengage. Thus, failure (versus success) should moderate the relationship between autonomous motivation for collective action and opinion-based group identification. In recent research, identification as a supporter of global poverty reduction was shown to mediate within-person changes in the relationship between autonomous motivation and collective action, but there was weak evidence for the reverse (Yip et al., 2024). We therefore expect that autonomous motivation will sustain collective action through failure via its effects on opinion-based group identification.

## Effect of Autonomous Motivation May Differ When Outcome Framing Is Specific Versus Broad

Thus far, we have argued that the effects of autonomous motivation on collective action and identification will differ after experiencing failure versus success. We propose that this effect may be further qualified by the framing of movement goals as specific or broad; Figure 1 depicts this proposed three-way interaction. We conceptualize specific goals as those that focus on the outcome of a singular campaign (e.g., to pass or block legislation change, achieve a desired outcome in an election). In contrast, broad goals refer to the bigger picture issue that a movement is seeking to progress (e.g., to achieve gender equality, combat climate change). We incorporated the theorizing around framing into the design for Study 3 to address findings of Studies 1–2.

**Figure 1.**
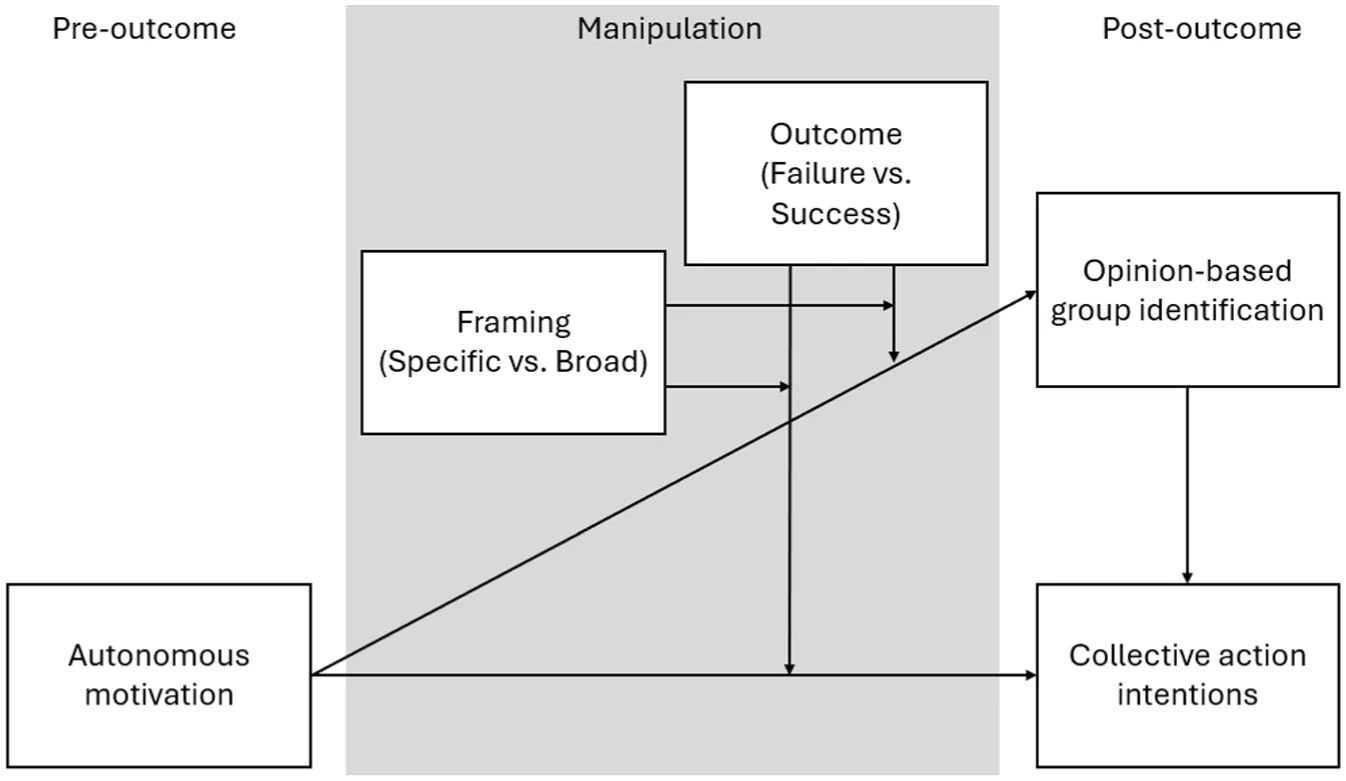
Moderated Mediation: Indirect Effect of Autonomous Motivation on Collective Action Intentions via Opinion-Based Group Identification, Moderated by Outcome (Studies 1–3) and Framing (Study 3).

Autonomous motivation may be more strongly associated with collective action after a *specific failure* than a *broad failure.* Research on goal striving suggests that the more important it is to an individual to achieve an outcome, the more strongly they experience outcome-related emotions (Pekrun & Stephens, 2010). It may be that a specific failure—given its concrete and proximal nature—may elicit strong perceptions of illegitimacy and feelings of anger, particularly for highly committed supporters, and thus may strengthen the relationship between autonomous motivation and collective action (Tausch & Becker, 2013). However, when the wider movement is failing, supporters may view the situation as stable and unchangeable, leading even the most highly invested supporters to disengage. Autonomous motivation may therefore be more effective to sustain action in a context of specific failure than broad failure.

Conversely, autonomous motivation may be more strongly associated with collective action after a *broad success* than a *specific success.* Research on psychological distance and goal striving suggests that positive feedback is interpreted differently in the context of psychologically distant or proximal goals (Fishbach et al., 2010). When people receive positive feedback for concrete, specific goals, they infer that they have made sufficient progress and can reduce their efforts. However, when pursuing psychologically distant, superordinate goals, positive feedback does not signal that sufficient progress has been made as the goal is still far from reach; instead, it highlights the individual’s commitment to the goal and increases expectations of goal attainment, and thus drives continued action (Fishbach et al., 2010). Thus, the more people autonomously value the cause, the more success should re-affirm their commitment to collective action and encourage further pursuit. Autonomous motivation may therefore be more effective to sustain action in the context of broad than specific success.

## The Present Research

In three studies (see Table 1), we tested the proposition that autonomous motivation would sustain opinion-based group identification and collective action intentions after failure. These studies were conducted in the context of diverse social issues, allowing us to test the generalizability and robustness of our findings across multiple issues and intergroup contexts. We first tested whether the indirect effect of autonomous motivation on collective action, via identification, would be moderated by experiencing failure or success (see Figure 1). We pre-measured autonomous motivation before failure/success occurred (Study 1) or was manipulated (Studies 2–3); we then measured identification as a supporter of the movement and collective action intentions. In Study 3, we tested the additional proposition that the effects of autonomous motivation on identification and collective action intentions would differ in the context of a specific campaign versus broad movement failure/success. Our theorizing around the specific/broad framing was developed and incorporated into the model after data analysis for Studies 1 and 2 was complete and thus is not considered in the design and hypotheses for the first two studies.

**Table 1.** Overview of the Present Research.

Category	Study 1	Study 2	Study 3
Issue	Marriage equality	Various social issues	Climate change
Country	Australia	Australia	USA
Year	2017	2021	2024
Focus	Outcome (success, failure)	Outcome (success, failure, control)	Outcome (success, failure) Framing (specific, broad)
Design	Quasi-experiment around the outcome of the Australian marriage equality plebiscite	Experimental manipulation of outcome	Experimental manipulation of outcome and framing
Pre-registration	No	Yes, after data collection, before analysis	Yes, before data collection

## Study 1

To examine how the effects of autonomous motivation on identification and collective action differ based on movement outcomes, we needed a context where there would be a clear distinction between a group who experienced failure and one who experienced success. We used data collected in 2017 when Australia held a nationwide plebiscite (billed as a survey) where citizens could opt-in to vote on whether marriage equality should be legalized. The plebiscite would reveal a percentage of “yes” and “no” responses, with the majority decision to be actioned by the government, meaning that there would be a clear success for one group and failure for the other. As there was a majority of “yes” votes (61.6%), marriage equality was legalized by the end of 2017; we therefore consider “yes” campaign supporters as experiencing success and “no” campaign supporters as experiencing failure.

We employed a quasi-experimental design to examine the effects of autonomous motivation pre-outcome (during the voting period) on opinion-based group identification and collective action intentions at post-outcome (after the results of the vote were announced). We compared the nature and strength of this effect between those who experienced failure (“no” supporters) and those who experienced success (“yes” supporters). We expected that autonomous motivation prior to the outcome would positively predict identification (H1) and collective action (H2) post-outcome, and there would be an indirect effect of autonomous motivation on action via identification (H3). We expected that this indirect effect would be stronger for those who experienced failure than for those who experienced success (H4).

### Openness and Transparency

Data were collected at three timepoints (Time 1: during the voting period, Time 2: immediately after the outcome of the plebiscite was announced, and Time 3: six weeks later). We used a subset of respondents, including only those who indicated a base level of engagement in action. Specifically, participants were only shown the motivation measures at each timepoint if they indicated that they had taken at least one action in the prior 6 weeks. We therefore only included those who took at least one action at Time 1 (but who did not necessarily take action at Time 2 or 3) and did not include Time 3 measures in this analysis. We did not pre-register specific hypotheses; the current analyses are therefore exploratory. Additional information including means and correlations for key variables can be found in the Supplementary Files.

## Method

### Participants

We analyzed a dataset consisting of 839 members of the Australian public who were eligible to vote in the marriage equality plebiscite (citizens over the age of 18), recruited via Qualtrics. Among these, 453 were excluded as they had taken no action at Time 1 and therefore did not complete the motivation measures, and a further 200 were excluded as they did not participate at Time 2. The final sample (*N* = 186) included 111 “yes” supporters and 75 “no” supporters. The average age was 59.31 (*SD* = 13.62). The sample was 45.2% female, 53.8% male, and 1.1% selected “prefer to self-describe.” Over a third of participants (36.5%) had completed a bachelor’s degree or higher. The majority of the sample (91.9%) identified as straight/heterosexual. We performed a sensitivity analysis in G*Power 3.1 (Faul et al., 2009) which indicated that with 186 participants, the minimum detectable effect size for the interaction at 80% power would be Cohen’s *f*^2^ = .04, assuming a two-tailed test and an alpha of .05. This sample size would provide 48% power to detect a small effect (*f*^2^ = .02) and >99% power to detect a medium effect (*f*^2^ = .15).

### Procedure and Measures

After providing informed consent, participants were asked to indicate whether they supported or opposed marriage equality and were subsequently shown measures relevant to their position. All items were measured on a scale from 1 (strongly disagree) to 7 (strongly agree), and responses were averaged to create a composite score for each variable. The full questionnaire can be viewed at https://osf.io/3gvsn/; relevant measures are described below.

#### Motivation

Participants were asked to indicate the extent to which they took the above actions because of a range of motives. There were four items for autonomous motivation (α = .89; e.g., “These actions are a fundamental part of who I am”) adapted from Weinstein and Ryan (2010). Two items were used to measure introjected regulation (e.g., “I would feel guilty if I didn’t do it”) and external regulation (e.g., “I do it to avoid being criticized”), respectively.

#### Opinion-Based Group Identification

Six items measuring identification with people who oppose/support marriage equality (α = .90; e.g., “I often think about the fact that I oppose [support] marriage equality”) were adapted from Leach et al. (2008) and Postmes et al. (2013).

#### Collective Action Intentions

Participants indicated the extent to which they intended to engage in five actions to either support or oppose marriage equality, aligning with their stated positions (α = .86; e.g., post on social media, join a peaceful protest).

## Results

To test the proposed moderated mediation (Figure 1), we used PROCESS version 4.3 (Hayes, 2017) in SPSS (Model 8) to examine whether the indirect effect of autonomous motivation on collective action intentions, via opinion-based group identification, would differ for those who experienced failure (effect coded 1) versus success (effect coded –1). We used the Time 1 measures of autonomous motivation and Time 2 measures of identification and collective action. We used 5,000 bootstrapped samples in the analyses of the conditional indirect effects for all studies. Identification and action intentions at T1 were entered as covariates, controlling for prior levels of these variables.

Consistent with H1, autonomous motivation positively predicted identification at Time 2 (see Table 2). Contrary to H2, autonomous motivation had no effect on action. However, there was a positive indirect effect of autonomous motivation on collective action, mediated by opinion-based group identification, consistent with H3 (*b* = 0.12, 95% CI = [0.01, 0.23], *SE* = .06). We did not find any significant interaction effects of autonomous motivation and outcome on the dependent variables, and the index of moderated mediation was not significant. Thus, we did not find support for H4.

**Table 2.** Test of the Effects of Autonomous Motivation on Collective Action Intentions via Opinion-Based Group Identification, Moderated by Failure.

	Opinion-based group identification T2			Collective action intentions T2		*p*
Predictor	*b* [95% CI]	*SE*	*p*	*b* [95% CI]	*SE*	
Outcome (failure vs. success)	–0.42 [–1.24, 0.41]	0.42	.319	–0.58 [−1.47, 0.31]	0.45	.203
Opinion-based group identification T2	-	-	-	**0.53 [0.38, 0.69]**	**0.08**	**<.001**
Autonomous motivation T1	**0.24 [0.08, 0.40]**	**0.08**	**.004**	0.07 [−0.11, 0.24]	0.09	.470
Outcome*Autonomous motivation T1	0.08 [−0.06, 0.21]	0.07	.265	0.11 [−0.04, 0.25]	0.08	.163
Opinion-based group identification T1	**0.56 [0.41, 0.71]**	**0.08**	**<.001**	**−0.25 [−0.44, −0.07]**	**0.09**	**.008**
Collective action intentions T1	0.06 [−0.04, 0.16]	0.05	.262	**0.78 [0.67, 0.89]**	**0.06**	**<.001**

*Note*. Index of moderated mediation = .08 [-.07, .24], *SE* = 0.08. Bold text denotes that confidence interval does not include zero.

We examined the simple slopes on an exploratory basis and found that there was a positive indirect effect of autonomous motivation on collective action for participants who experienced failure; see Table 3. There were no significant effects of autonomous motivation for those who experienced success. However, these effects were not different from each other as there was no evidence of an interaction, as reported above.

**Table 3.** Simple Slopes Analysis of the Effects of Autonomous Motivation by Outcome.

	Opinion-based group identification T2		Collective action intentions T2		Indirect effect	*SE*
Outcome	*b* [95% CI]	*SE*	*b* [95% CI]	*SE*	*b* [95% CI]	
Failure	**0.32 [0.08, 0.55]**	**0.12**	0.17 [−0.09, 0.43]	0.13	**0.17 [0.01, 0.34]**	**0.08**
Success	0.16 [−0.02, 0.34]	0.09	−0.04 [−0.24, 0.16]	0.10	0.09 [−0.03, 0.20]	0.06

*Note*. Bold text denotes that the confidence interval does not include zero.

### Robustness Check

We also conducted a robustness check to examine whether effects differed once the role of controlled motivation was accounted for. We tested an identical model with controlled forms of motivation (introjected and external regulation) entered as covariates to isolate the effect of autonomous motivation. The pattern of results was unchanged and so our reporting focuses on the simpler model without covariates.

### Supplementary Analyses

To isolate the effects of autonomous motivation as a uniquely important predictor of resilience after setbacks, we also conducted exploratory analyses examining the role of introjected and external regulation, respectively. We conducted these Supplementary analyses in all three studies and found no effects of introjected or external regulation except in Study 3; we report these analyses in the Supplementary Materials.

## Discussion

The data suggested that autonomous motivation prior to an experience of success or failure was associated with opinion-based group identification and, in turn, collective action intentions after the campaign had ended. Thus, autonomous motivation is associated with persistent action; however, contrary to our predictions, there was no evidence of moderation, suggesting that the effects of autonomous motivation are not more powerful after failure.

A limitation of Study 1 is that we did not manipulate outcomes and instead used two naturally occurring groups with distinct characteristics, attitudes, and predispositions. The failure group were opponents of marriage equality advocating to maintain the conservative status quo, while the success group were supporters seeking progressive change. There were also key differences in the tactics used, the leaders within the movements, and the political discourse around the two groups, resulting in many confounds and contextual factors that were not controlled for within this quasi-experimental approach. We addressed these issues in Study 2 using an experimental design.

## Study 2

Study 1 provided preliminary evidence that autonomous motivation had a positive effect on identification and collective action after experiencing failure, but did not suggest that this differed from those who experienced success. In Study 2, we tested our hypotheses with an experimental design where outcomes were manipulated. To maximize ecological validity, we primed participants in real social movements to focus on either success, failure, or neither. Furthermore, while Study 1 was tied to a specific context (the marriage equality plebiscite), in Study 2, we recruited Australian citizens who had taken some political action and allowed them to select a movement of their choosing from a list (see Table 4). Our sample comprised people who reported having engaged in at least one form of collective action other than voting in the last year (voting in general elections is compulsory in Australia and is therefore not a reliable indication of political engagement). We also included a control group to test whether the effects of autonomous motivation may increase or decrease following the manipulation of outcomes, compared to a baseline. The control group may be influenced by participants’ pre-existing perceptions of success/failure, but these were not made salient.

**Table 4:** Number of Participants Who Selected Each Cause.

Cause	Frequency	Cause	Frequency
Climate change	63	Fee increases at Australian universities	2
Animal rights	43	Anti-immigration	2
Women’s rights	11	Refugee rights	3
Pro-LGBT rights	4	Pro-religious freedom	1
Black Lives Matter	23	Anti-lockdown	2
All Lives Matter	4	Anti-vaccination	2
Pro-life	4	Pro-vaccination	10
Pro-choice	0	Other	8

We hypothesized that pre-measured autonomous motivation would positively predict identification (H1) and collective action (H2) post-manipulation. There should be an indirect effect of autonomous motivation on action via identification (H3) and, as such, identification would positively predict action (H4). We predicted that the effects in H1–3 would be stronger in the failure condition than the control condition (H5), and stronger in the control condition than the success condition (H6).

### Openness and Transparency

Pre-registration for Study 2 occurred after data collection but before examining the data; see https://osf.io/72xgz/?view_only=e1d4fc8071494a7383446ad2a96ad892 for registration and https://osf.io/8ryk6/?view_only=c76508adba144666b96d34ceab7096c8 for all materials. Deviations from the pre-registration are declared transparently below. We implemented a 2 (success: yes/no) × 2 (failure: yes/no) × 2 (time: pre/post) design where participants were primed to think about examples of their movement succeeding, failing, neither, or both; we omitted the participants who received both manipulations (“combined” condition), in line with our pre-registration, but report an analysis using this group in the Supplementary Materials.

## Method

### Participants and Design

We utilized an existing dataset of 620 participants who were recruited at Time 1, and 305 who also completed the survey at Time 2. We removed 18 participants with missing data on key variables, one participant who wrote a nonsensical answer to the manipulation, and one who selected “I have just clicked through” when asked if they had taken the survey seriously (per Aust et al., 2013). We did not pre-register any further exclusions. However, at the start of the survey, participants were instructed to choose whichever cause they had been most actively involved with in the past 12 months. We assumed that most participants would choose the same cause at both timepoints, but 103 did not. We excluded these participants as we could not meaningfully compare the effects of autonomous motivation in the context of one cause or issue (at Time 1) on subsequent responses to other issues (at Time 2). We therefore had a total of 182 valid responses; after removing participants in the “combined” condition, the final sample included 137 participants. The mean age was 45.67 (*SD* = 17.03), and 63.5% of the sample were female. The sample was highly educated, with 48.1% indicating that they had completed a bachelor’s degree or higher. A sensitivity analysis using G*Power suggested that the minimum detectable effect size for the interaction at 80% power would be Cohen’s *f*^2^ = .06. The sample size would provide 38% power to detect a small effect and 99% power to detect a medium effect.

### Procedure

Participants were recruited via Qualtrics Panels. At Time 1, after providing informed consent, participants were shown a list of popular causes and asked to indicate which ones they had been actively involved with in the last year. They could also write in up to three causes of their choosing. Participants were then asked to select one cause that they had been most involved in. Table 4 displays the list of choices and the number of participants who selected each option. Subsequently, we used piped text to insert the selected cause in all remaining questions in the survey (where relevant). Participants then completed the dependent measures.

Participants completed the Time 2 questionnaire 2–5 weeks after Time 1, allowing us to obtain a measure of motivation without creating demand characteristics for the experimental phase. The Time 2 survey was nearly identical except after selecting their chosen cause, participants were shown the outcome manipulation (except those in the control condition, who proceeded directly to the dependent measures). At the end of the survey, we also added a manipulation check measuring participants’ perceptions of their movement’s failure and success.

### Manipulation

We primed participants to consider their chosen movement’s successes or failures by showing them this prompt: “In the box below, please describe and reflect on at least 3 ways the [chosen cause] movement has failed [been successful] in the last year. This could include failures [successes] related to achieving your central goals, increasing public awareness of the cause, swaying stakeholders’ opinions, and/or building an oppositional movement.”

Participants in the “success” group only wrote about success (*n* = 49); participants in the “failure” group only wrote about failure (*n* = 45); those in the “control” condition were not shown either prompt and did not complete a writing task (*n* = 43).

### Measures

#### Manipulation Check

There was one manipulation check of failure, and one of success: “To what extent do you agree that: The [chosen cause] movement has experienced failures [successes] within the last year” rated on a 1 (not at all) to 7 (very much) scale.

#### Motivation

The same items from Study 1 were used to measure autonomous motivation (α = .85), introjected regulation and external regulation.

#### Opinion-Based Group Identification

Participants were asked to what extent they identified with supporters of their chosen cause using the same adapted items from Study 1 (α = .90 e.g., “I identify with other members of the [chosen cause] movement.”)

#### Collective Action Intentions

Participants indicated the likelihood that they would engage in 10 collective actions in future on a 1 (not at all likely) to 7 (very likely) scale (e.g., attend a public meeting, sign an online/digital petition; α = .84).

## Results

### Manipulation Check

A one-way ANOVA in SPSS demonstrated partial support for the effectiveness of the success manipulation, *F*(2, 134) = 4.73, *p* = .010, η^2^= .07. Participants in the success condition (*M* = 5.00, *SD* = 1.50) scored higher on the success manipulation check than participants in the failure condition (*M* = 4.18, *SD* = 1.35, *p* = .012, Cohen’s *d* = 0.57), which did not significantly differ from the control condition participants (*M* = 4.86, *SD* = 1.23, *p* = .878). There were no differences in mean scores between conditions on the failure manipulation check item (*M* = 4.14–4.58, *SD* = 1.33–1.66), *F*(2, 134) = 1.21, *p* = .302, η^2^ = .02. All groups scored above the midpoint of the scale for both success and failure checks. We therefore concluded that the success manipulation had functioned partially as intended, but we did not effectively manipulate failure.

### Moderated Mediation

We used the same analysis as in Study 1 to test the indirect effect of autonomous motivation (T1) on collective action intentions (T2), via opinion-based group identification (T2). Identification and action intentions at T1 were entered as covariates to account for stability over time. We used the multi-categorical option in PROCESS to specify the moderating variable using effect coding; failure (effect coded 1) was compared with control (effect coded −1) to test H3, and success (effect coded 1) was compared with control (effect coded −1) to test H4. As in Study 1, we conducted a robustness check by re-running the model with the controlled motivations (introjected, external) entered as covariates. The pattern of results was the same and thus we report the simpler model (as per our pre-registration).

A summary of effects is presented in Table 5. Autonomous motivation (T1) positively predicted identification (T2), providing support for H1. There was no direct effect of autonomous motivation on action intentions (T2), inconsistent with H2, but there was a significant indirect effect via opinion-based group identification (H3; *b* = 0.10, 95% CI = [0.03, 0.20], *SE* = .04). There was a positive effect of identification on action (H4).

**Table 5.** Test of the Effects of Autonomous Motivation on Collective Action Intentions via Opinion-Based Group Identification, Moderated by Failure and Success.

	Opinion-based group identification T2			Collective action intentions T2		*p*
Predictor	*b* [95% CI]	*SE*	*p*	*b* [95% CI]	*SE*	
Failure (vs. control)	0.57 [−0.62, 1.77]	0.60	.343	−0.02 [−1.27, 1.22]	0.63	.970
Success (vs. control)	−1.11 [−2.34, 0.12]	0.62	.077	−0.37 [−1.67, 0.92]	0.65	.569
Opinion-based group identification T2	-	-	-	**0.39 [0.21, 0.57]**	**0.09**	**<.001**
Autonomous motivation T1	**0.26 [0.10, 0.42]**	**0.08**	**.002**	0.16 [−0.01, 0.34]	0.09	.070
Failure × Autonomous motivation T1	−0.11 [−0.33, 0.10]	0.11	.296	0.05 [−0.17, 0.28]	0.11	.631
Success × Autonomous motivation T1	0.19 [−0.02, 0.40]	0.11	.081	0.00 [–0.022, 0.23]	0.11	.988
Opinion-based group identification T1	**0.51 [0.36, 0.65]**	**0.07**	**<.001**	−0.08 [−0.26, 0.09]	0.09	.339
Collective action intentions T1	0.09 [−0.04, 0.21]	0.06	.165	**0.40 [0.27, 0.52]**	**0.07**	**<.001**

*Note*. Index of moderated mediation for failure = −0.04, 95% CI = [−0.14, 0.03], *SE* = 0.04. Index of moderated mediation for success = 0.07, 95% CI = [−0.02, 0.19], *SE* = 0.05. Bold text denotes that confidence interval does not include 0.

H5–6 addressed the moderated mediation (see Table 5). Contrary to H5–6, there were no significant interactions between autonomous motivation and failure, or success, on the dependent variables. All the indices of moderated mediation were non-significant.

As with Study 1, we examined the simple slopes on an exploratory basis (see Table 6). The indirect effect of autonomous motivation on collective action intentions, via opinion-based group identification, was significant in the success condition but not in the failure or control conditions. Thus, the simple slopes did not provide clear evidence that autonomous motivation sustained action after failure.

**Table 6.** Simple Slopes Analysis of the Effects of Autonomous Motivation by Outcome.

	Opinion-based group identification		Collective action intentions		Indirect effect	*SE*
Outcome	*b* [95% CI]	*SE*	*b* [95% CI]	*SE*	*b* [95% CI]	
Failure	0.15 [−0.12, 0.41]	0.14	0.22 [−0.06, 0.50]	0.14	0.06 [−0.04, 0.15]	0.05
Success	**0.45 [0.18, 0.73]**	**0.14**	0.17 [−0.13, 0.47]	0.15	**0.18 [0.03, 0.36]**	**0.08**
Control	0.18 [−0.09, 0.45]	0.14	0.11 [−0.17, 0.39]	0.14	0.07 [−0.03, 0.18]	0.05

*Note*. Bold text denotes that confidence interval does not include zero.

## Discussion

Consistent with Study 1, autonomous motivation predicted opinion-based group identification and, in turn, collective action at Time 2. Once again, failure and success did not significantly moderate this relationship. However, when examining the simple slopes, we found some evidence of an opposite pattern to Study 1; in Study 2, the indirect effect of autonomous motivation on action via identification was significant for those in the success condition, but not the failure condition. These findings were unexpected and inconsistent with the proposition that autonomous motivation should be associated with sustained collective action after failure.

There were some key differences in the contexts in which Studies 1 and 2 were conducted. Study 1 involved a concrete, specific instance of failure (the outcome of the marriage equality plebiscite), while Study 2 referred to more general or abstract perceptions of a social movement experiencing failures “within the past year.” For example, many Study 2 participants wrote about not being heard, or insufficient funding for their chosen causes. Thus, autonomous motivation may be important to sustain action after a *specific* instance of failure, while the overall movement might still be making progress. In contrast, autonomous motivation may be less effective at sustaining action when reflecting on *broader* movement failures, as they may signal that the situation is unchangeable and collective action will be ineffective.

There were also some aspects of Study 2 that made it difficult for us to control or fully understand how participants interpreted success and failure. First, as participants were allowed to choose their preferred cause and generate their own examples of failure and success, there was likely to be a high degree of variability related to the different contexts and events on which participants reflected (see Table 4). Second, our manipulation check results suggested that we did not effectively manipulate failure or success relative to the control group. Finally, both Study 1 and 2 were underpowered to detect small effects; these issues were addressed in Study 3.

## Study 3

We conducted a final experiment to address the limitations of the prior studies. To this end, we recruited self-identified supporters of one cause: the climate movement. We chose the climate movement as it was the cause most commonly selected by participants in Study 2 (see Table 4), and provided a context where we could believably manipulate success and failure of both a specific and broad goal without making implausible claims. We provided text stating that the climate movement is failing or succeeding, including examples and quotes from an expert, to create a more consistent manipulation. We also manipulated the framing of failure and success as applying either to a specific, concrete outcome (the campaign to obtain a commitment to phaseout of fossil fuels at the 2023 United Nations Climate Change Conference) or to a broader, abstract movement (the climate movement more generally). Doing so allowed us to systematically explore whether the framing of the movement outcomes may explain the presence of the focal effects in the failure condition in Study 1 but not Study 2.

We pre-registered hypotheses for Study 3. Pre-registration occurred prior to data collection (see https://osf.io/8ybrq/?view_only=e1628a170e044e2cbcb501fb0ddba4e1 for registration, and https://osf.io/8ryk6/?view_only=c76508adba144666b96d34ceab7096c8 for all materials). We expected that there would be a three-way interaction between outcome, framing, and autonomous motivation. As in Studies 1–2, autonomous motivation should positively predict opinion-based group identification (H1) and collective action intentions (H2). However, when reflecting on the outcome of a specific campaign, this relationship should be stronger for those in the failure (vs. success) condition (H3). Conversely, when reflecting on the outcomes of the broader movement, this relationship should be stronger in the success (vs. failure) condition (H4). Finally, we predicted that the effects described in H3–4 would be mediated by opinion-based group identification (H5).

## Method

### Participants and Design

An a priori power analysis using G*Power indicated that at least 395 participants were required to detect a three-way interaction with 80% power and a small effect size (*f*^2^ = .02). Thus, we initially recruited 404 US-based supporters of climate action. The choice to switch to a US sample was for practical reasons, as there is a large MTurk participant pool in the US. Per our pre-registration, we removed four participants for failing an attention check and 23 who failed a comprehension check (described below). The final sample included 377 participants. A sensitivity analysis suggested that this slightly reduced sample size would provide 78% power to detect a small effect. The average age was 43.70 (*SD* = 13.17), and the sample was 45.5% female, 53.6% male, and 0.5% non-binary/other. The sample was highly educated, with 71.3% possessing a bachelor’s degree or higher. The majority (85.7%) reported that they had taken at least one action to support the climate movement in the prior 12 months.

The experiment employed a 2 (outcome: failure, success) × 2 (framing: specific, broad) design. Autonomous motivation was measured prior to the manipulation, while identification and action intentions were measured after. We employed a single-timepoint pre- and post-manipulation design to reduce the risk of attrition (per Studies 1 and 2).

### Procedure and Manipulations

We recruited participants through MTurk to participate in a 10 min survey on Qualtrics. We informed participants that we would award a 50c bonus payment if they demonstrated that they had paid attention to the materials. Only participants who provided informed consent and responded “yes” to the question “Do you support global action to combat climate change?” were eligible to participate. Eligible participants then completed measures of past action and motivation before being randomly allocated to one of four conditions: specific-success, specific-failure, broad-success, and broad-failure. They were shown a short text about the current state of the climate movement with different content depending on the allocated condition.

Participants in the *specific* conditions read a text about the 2023 United Nations Climate Change Conference (known as COP28) which suggested that the meeting was a *success* as “for the first time ever, the final agreement between participating countries included acknowledgment of the need to transition away from fossil fuels,” or a *failure* because “despite the efforts of campaigners, the final agreement between participating countries failed to include any commitment to phase out fossil fuels.” While the information in both passages was factual, we varied whether this was portrayed as a success or failure by presenting transition or phaseout of fossil fuels as the campaign’s goal.

In the *broad* conditions, participants read about “the climate movement” rather than any specific event or campaign. The passages stated that the movement is *succeeding* because “investment in renewable energy continues to increase every year, and major falls in the cost of solar and wind energy suggest that the end of fossil fuels is near,” or *failing* because “carbon emissions continue to increase every year, and the approval of hundreds of new oil and gas projects suggests that the end of fossil fuels is nowhere in sight.” The full passages are presented in the Supplementary Materials. In each condition, participants were kept on the page for at least 30 s before they could move forward in the survey. Participants then completed comprehension and manipulation checks, followed by measures of opinion-based group identification and collective action intentions, a range of other exploratory measures, and demographic questions.

### Measures

#### Comprehension Check

Participants were asked whether they had read about COP28 or the climate movement more generally.

#### Manipulation Check

Participants were asked to indicate whether the article they read had described goals that were “very real and concrete” or “very abstract and distant,” on a sliding scale from 0 to 100, with higher scores indicating greater abstractness. They were also asked whether in their opinion, COP28/the climate movement more generally (depending on their assigned condition) was a success or a failure, on a similar 0–100 sliding scale with higher scores indicating greater perceived failure.

#### Motivation

We used the same measures of autonomous motivation (α = .85), introjected regulation (*r* = .62), and external regulation (*r* = .60).

#### Opinion-Based Group Identification

We used the same six items as previously, with a specific focus on identification with supporters of action to combat climate change (α = .90).

#### Collective Action Intentions

We asked participants to indicate their likelihood of taking six actions in the next twelve months (e.g., sign a petition, contact a politician, α = .86). We also measured radical action intentions, and include an analysis of this variable in the Supplementary Files.

#### Attention check

Finally, we asked participants: “Which of these topics was discussed?” We granted the 50c bonus to participants who selected “climate change” out of four possible topics *and* correctly answered the comprehension check. Those who failed these checks (*n* = 27) were removed from analyses.

## Results

### Manipulation Checks

We conducted factorial ANOVAs to determine the effectiveness of the outcome and framing manipulations (see Tables 7 and 8 for cell means).

**Table 7.** Cell Means on Outcome Manipulation Check.

Condition	Specific	Broad	Total
Failure	82.08 (22.24)	62.39 (21.82)	72.75 (24.09)
Success	25.59 (24.70)	39.39 (21.48)	32.19 (24.16)
Total	54.26 (36.75)	50.96 (24.47)	52.69 (31.51)

*Note.* Higher scores indicate greater perceived failure.

**Table 8.** Cell Means on Framing Manipulation Check.

Condition	Specific	Broad	Total
Failure	42.95 (28.96)	48.00 (29.74)	45.34 (29.36)
Success	33.43 (28.40)	32.71 (24.19)	33.09 (26.41)
Total	38.24 (29.01)	40.40 (28.12)	39.26 (28.57)

*Note.* Higher scores indicate greater perceived abstractness.

#### Outcome Manipulation

As expected, there was a main effect of outcome wherein participants in the failure condition scored higher on the failure manipulation check than those in the success condition, *F*(1, 372) = 289.25, *p* < .001, η_p_^2^ .44. Unexpectedly, there was also an interaction between outcome and framing, *F*(1, 372) = 51.37, *p* < .001, η_p_^2^ = .12. Participants in the specific-failure condition rated failure as higher than those in the broad-failure condition, *t*(188) = 6.15, *p* < .001, Cohen’s *d* = .89. Participants in the broad-success condition rated failure as higher than those in the specific-success condition, *t*(184) = −4.05, *p* < .001, Cohen’s *d* = 0.60. Both failure conditions scored above the midpoint, while both success conditions scored below the midpoint. Thus, the manipulation of outcome was effective; however, both manipulations were stronger when the framing was specific (i.e., failure was higher in the failure condition, but success was higher in the success condition). It may be that outcomes are perceived as clearer and more concrete in the context of a specific campaign than a wider, ongoing movement.

#### Framing Manipulation

There was no main effect of framing, *F*(1, 373) = 0.56, *p* = .453. Participants in both specific and broad conditions rated the article’s goals as closer to “very real and concrete” than “very abstract and distant” but were nevertheless close to the midpoint. Thus, the effects of the framing manipulation reported below may be attributed to characteristics of the manipulation other than psychological distance of movement goals. For instance, broad movements are ongoing and their outcomes are not yet set in stone, while specific movements are singular events with clearer outcomes (see further discussion below). Unexpectedly, there was a main effect of outcome, *F*(1, 373) = 18.52, *p* < .001, η_p_^2^ = .05. Participants in the failure conditions rated the goals as more abstract and distant than those in the success conditions. The evidence in relation to the failure and framing manipulations suggested that it is difficult to orthogonally manipulate outcomes without also manipulating abstraction.

### Hypothesis Testing

Per our pre-registration, we conducted these analyses with and without introjected and external regulation added as covariates. Key findings related to the hypotheses remained the same. However, unlike Studies 1–2, there were significant effects of introjected and external regulation on the dependent variables (Table 9), so we reported the results of the analysis that included the covariates to ensure our reported effects are specific to autonomous motivation.

**Table 9.** Test of the Effects of Autonomous Motivation on Collective Action Intentions via Opinion-Based Group Identification, Moderated by Outcome and Framing.

	Opinion-based group identification			Collective action intentions		*p*
Predictor	*b* [95% CI]	*SE*	*p*	*b* [95% CI]	*SE*	
Autonomous motivation	**0.51 [0.41, 0.61]**	**0.05**	**<.001**	**0.13 [0.001, 0.25]**	**0.06**	**.048**
Outcome (failure vs. success)	−0.17 [−0.56, .023]	0.20	.404	0.27 [−0.17, 0.72]	0.23	.229
Framing (specific vs. broad)	−0.24 [−0.64, 0.15]	0.20	.229	0.17 [−0.28, 0.62]	0.23	.452
Opinion-based group identification	-	-	-	**0.63 [0.52, 0.75]**	**0.06**	**<.001**
Outcome*Autonomous motivation	0.02 [−0.05, 0.10]	0.04	.539	−0.04 [−0.12, 0.05]	0.04	.381
Framing*Autonomous motivation	0.03 [−0.04, 0.11]	0.04	.363	−0.05 [−0.14, 0.03]	0.04	.222
Outcome*Framing	**−0.44 [−0.83, −0.04]**	**0.20**	**.029**	0.37 [−0.08, 0.81]	0.23	.109
Outcome*Framing*Autonomous motivation	**0.08 [0.01, 0.16]**	**0.15**	**.026**	−0.06 [−0.14, 0.03]	0.04	.189
Introjected regulation	0.07 [0.00, 0.15]	0.04	.056	**0.19 [0.10, 0.28]**	**0.04**	**<.001**
External regulation	0.04 [−0.03, 0.10]	0.03	.302	0.06 [−0.02, 0.14]	0.04	.131

*Note*. Index of moderated moderated mediation = 0.05 [−0.02, 0.13], *SE* = 0.06. Index of moderated mediation (by outcome) in specific conditions = 0.07 [−0.002, 0.14], *SE* = 0.04. Index of moderated mediation (by outcome) in broad conditions = −0.04 [−0.15, 0.09], *SE* = 0.06. Bold text denotes that confidence interval does not include zero.

We first tested the interaction between autonomous motivation, outcome, and framing on opinion-based group identification (see Table 9). We used effect coding of failure (1) and success (−1), and specific (1) and broad (−1) framing. Autonomous motivation positively predicted identification, providing support for H1. There was a significant three-way interaction (see Figure 2). Simple interaction tests revealed that in the specific condition, the effect of autonomous motivation on identification was stronger when participants read about failure, vs. success; *b* = 0.11, *F*(1, 367) = 4.42, *p* = .036, thereby supporting H3. However, in the broad conditions, the strength of the motivation-identification link was the same regardless of whether participants read about a failure or success of the climate movement, *b* = −0.06, *F*(1, 367) = 1.21, *p* = .271. Thus, H4 was not supported. Autonomous motivation predicted identification in all conditions, but this effect was stronger after specific failure (see Table 10).

**Figure 2.**
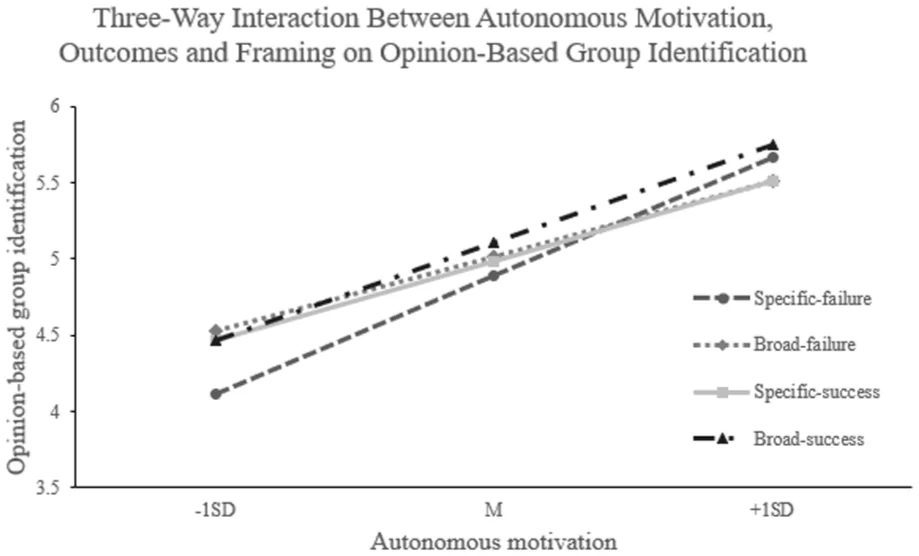
Three-Way Interaction Between Autonomous Motivation, Outcomes, and Framing on Opinion-Based Group Identification.

**Table 10:** Simple Slopes Analysis of the Effects of Autonomous Motivation by Condition.

		Opinion-based group identification		Collective action intentions		Indirect effect	
Framing	Outcome	*b* [95% CI]	*SE*	*b* [95% CI]	*SE*	*b* [95% CI]	*SE*
Specific	Failure	**0.65 [0.50, 0.80]**	**0.08**	−0.02 [−0.21, 0.17]	0.10	**0.41 [0.27, 0.54]**	**0.07**
	Success	**0.44 [0.28, 0.59]**	**0.08**	0.17 [−0.01, 0.35]	0.09	**0.28 [0.14, 0.41]**	**0.28**
Broad	Failure	**0.41 [0.25, 0.58]**	**0.08**	**0.20 [0.004, 0.39]**	**0.10**	**0.26 [0.08, 0.46]**	**0.10**
	Success	**0.54 [0.37, 0.71]**	**0.09**	0.16 [−0.04, 0.36]	0.10	**0.34 [0.21, 0.48]**	**0.07**

*Note*. Bold text denotes that confidence interval does not include 0.

We then tested the moderated moderated mediation model (H5), whereby the effect of autonomous motivation on action via identification is conditional upon specific failure (PROCESS Model 12). There was a positive direct effect of autonomous motivation on collective action intentions (H2), and an indirect effect via identification (*b* = 0.32, 95% CI = [0.23, 0.41], *SE* = .05). As expected, the indirect effect was stronger for those who read about a specific failure (vs. specific success); but stronger for those who read about a broad success (vs. broad failure; see Table 10). However, the index of moderated moderated mediation was not significant, and thus H5 was not supported.

## Discussion

Autonomous motivation was a positive predictor of identification regardless of movement outcomes which, in turn, indirectly predicted action intentions. As hypothesized, the effect of autonomous motivation on identification was stronger after experiencing a *specific campaign failure*, but not when the broader movement was failing. Thus, autonomous motivation appears particularly important to sustain identification after experiencing a specific moment of movement failure.

One complexity with Study 3 was that the manipulation check suggested that there were no differences in abstraction of the movement goals between the two framing conditions. Moreover, participants who read about a specific failure perceived failure more strongly than those who read about the climate movement failing more broadly; similarly, participants in the specific success condition rated success as higher than those in the broad success condition. This finding appears counter-intuitive, as one may expect that multiple or ongoing failures would be construed as more impactful relative to a single instance (e.g., Moghaddam et al., 1997). It may be that people construe failure and success more intensely when it relates to a past campaign or event with a clearly determined outcome compared to an ongoing movement. This may also explain why autonomous motivation was more strongly predictive of identification after a specific rather than broad failure. It is thus difficult to design an ecologically valid, orthogonal manipulation of outcomes and framing; this is a limitation of the present research.

Finally, the national context for Study 3 (US) differed from the first two studies (Australia). We considered these comparable samples given that Australia and the United States are both predominantly English-speaking Western, Educated, Industrialized, Rich, and Democratic (WEIRD) countries who share key cultural similarities (e.g., individualist, capitalist societies; see Henrich et al., 2010). Nevertheless, there are important differences; for instance, Australia’s democracy is stronger, but Americans score higher on political participation (Economist Intelligence Unit, 2024). Despite these differences, the theorized relationships are consistent across these two contexts.

## General Discussion

Social movements frequently experience failures, but little is known about what drives supporters to continue their efforts in the face of setbacks. We examined whether autonomous motivation would become particularly important to sustaining opinion-based group identification and collective action after experiencing failure. Table 11 presents a summary of the findings. In Studies 1–2, there was little evidence of moderation of the effects of autonomous motivation by failure. Exploratory simple slope analyses suggested that while the effect was present for the failure condition in Study 1, it was only present for the success condition in Study 2. In Study 3, we further investigated these inconsistent findings using an additional manipulation of framing of the movement as specific or broad. Study 3 revealed that the effect of autonomous motivation on identification was stronger after experiencing a failure, but only when failure was specific. Thus, the findings suggest that autonomous motivation is associated with greater resilience and continued action, but its role becomes more pronounced after experiencing a specific campaign failure. These findings are consistent with the DIME model, which argues that movement supporters respond more heterogeneously to failure than success (Lizzio-Wilson et al., 2021, 2024). However, this paper extends these findings to explain why some people maintain their support while others disengage following failure (Becker & Tausch, 2014); we suggest that autonomous motivation is a key factor which serves to maintain commitment. Furthermore, supporters’ responses to failure differ depending on whether they experience failure of an individual or multiple events, or even an entire movement.

**Table 11.** Summary of the Direct and Indirect Effects of Autonomous Motivation on Opinion-Based Group Identification and Collective Action by Condition Across Studies 1–3.

	Opinion-based group identification		Indirect effect (on collective action)	
Study	Failure	Success	Failure	Success
Study 1 (Specific)	✓		✓	
Study 2 (Broad)		✓		✓
Study 3 (Specific)	✓ ✓	✓	✓	✓
Study 3 (Broad)	✓	✓	✓	✓

*Note*. Check marks denote that the simple slopes indicated a positive, significant effect. Double checks indicate that effects were significantly stronger than other conditions in the same study.

Autonomous motivation consistently predicted higher opinion-based group identification, but this effect was more pronounced after experiencing a specific failure. The role of autonomous motivation in sustaining identification after failure is consistent with findings that when the group is low-status, less strongly committed individuals are more likely to disengage, while those who are committed to the group maintain identification regardless of its status (Blackwood & Louis, 2012). Thus, perhaps the effects of autonomous motivation were stronger after a *specific failure* because our manipulation check suggested that participants perceived failure as greater when it was specific to a past campaign than when it was ongoing. It may be that the effects of failure are felt more strongly in this context because it is acute and final—the failure has already occurred and the outcome cannot be changed—and thus autonomous motivation is needed to maintain identification. However, a broad movement failure is less clear as the outcome is not yet determined. Future research could interrogate participants’ reactions and examine qualitatively how failure or success is understood and subjectively experienced.

Autonomous motivation also indirectly sustained collective action via its effects on opinion-based group identification. This is consistent with Yip et al. (2024), and other findings that autonomously motivated individuals are more likely to continue engaging in goal-striving behavior after experiencing setbacks (e.g., Holding et al., 2017). However, in the context of collective action, these effects are primarily present when failure occurs in a specific event or campaign rather than when the movement is failing overall.

The research on individual goal pursuit suggests that when a goal is unattainable, autonomously motivated actors are particularly skilled at redirecting their efforts and re-engaging with a new goal that aligns with their identity and values (Smith & Ntoumanis, 2014), and are better at doing so the sooner they recognize that their original goal was unattainable (Ntoumanis et al., 2014). Thus, experiencing a specific failure may drive autonomously motivated actors to select new goals and redirect their efforts to the next campaign while still pursuing the same broader aims. However, it is not possible to redirect in the same way when the broader movement is failing. In addition, specific failure can be construed as a setback or obstacle, in the face of which autonomously motivated supporters may be more willing to expend additional effort (Holding et al., 2017; Leduc-Cummings et al., 2022). In contrast, broader movement failure may not be perceived as an obstacle but rather an indication that the movement more generally and its tactics are ineffective and making no progress; additional effort is therefore unlikely to overcome the movement’s failures, and those who are most strongly committed to the cause may pursue other tactics (Lizzio-Wilson et al., 2022; Louis et al., 2022).

### Limitations and Future Directions

One of the key limitations of the present research was that to examine the effects of motivation on collective action after actual (Study 1) or manipulated (Studies 2–3) success/failure of a social movement, we could only investigate effects on action intentions rather than actual behavior. Although collective action intentions and behavior are highly correlated (e.g., Fielding et al., 2008), some research on goal striving suggests that controlled motivation predicts *intentions* to expend effort after setbacks, but only autonomous motivation predicts *behavior* (Sheldon & Elliot, 1998). Our study designs with only 1–2 timepoints were unable to test the effects of motivation on actual behavior, but this is an important avenue for future research. However, a strength of the current research is its ability to adjudicate causal effects due to the use of mixed methodology examining participants’ responses to a real example of failure with high ecological validity (Study 1) and experimental manipulations with higher internal validity (Study 2–3). This mixed design allows us to rule out the potential confounds present in Study 1 (e.g., our sampling of ideologically opposing groups) but also provides evidence that the effects are present in a real-world context, when failure has actually occurred.

In all three studies, we conceptualized failure and success as binary outcomes of social movements. However, social movement scholarship suggests that these outcomes are not binary, as real-world movement outcomes rarely fit cleanly in one category or the other. Collective actors construe success and failure across multiple dimensions; for instance, forming new relationships or feeling empowered can be experienced as successes, even if a specific goal such as policy change is not achieved (Haiven & Khasnabish, 2013). Indeed, our findings suggested that it was not straightforward to manipulate success and failure. Future research could explore the link between motivation and sustained action in the context of these other outcomes and take a more nuanced approach to conceptualizing failure and success.

In the present research, we investigated the effects of autonomous motivation on social identification, and in turn collective action; however, we note that other causal orders are possible. Some research suggests that people are more likely to adopt and internalize norms of groups they identify with (e.g., Abrams et al., 1990), and that the strength of social identification may be a key determinant of how people respond to setbacks or threats (Ellemers et al., 1997; Spears et al., 1997). Thus, future research could investigate the role of social identification as a predictor of motivation or as a moderator of the effects of failure on collective action.

Research has now shown that autonomous motivation is a key predictor not only of sustained collective action over time (Yip et al., 2024) but also of continued action after facing setbacks. Future research should therefore investigate when and how autonomous motivation develops and explore strategies that would help foster autonomous motives among movement supporters. Relatedly, given that continued goal pursuit in the face of repeated failures may lead to burnout and political despair (Bird et al., 2024), future research should examine whether autonomous motivation can alleviate burnout and promote well-being (Sheldon et al., 2016) or, conversely, elicit rumination and difficulty disengaging from unattainable goals (Ntoumanis et al., 2014).

## Conclusion

Setbacks and failures are common in social movements that are often faced with challenges from opposing groups, and require continued efforts and resilience from their supporters to be effective. It is therefore important to understand the psychological factors that sustain collective action after failure, when supporters may be tempted to disengage. Our findings suggest that the more supporters hold an internalized commitment to the cause and value the social movement outcomes, the more likely they are to continue engaging in collective action after experiencing failures. Autonomous motivation is a uniquely effective driver of action in comparison to other forms of motivation that can help to promote resilience and long-term commitment among supporters.

## Supplemental Material

sj-docx-1-psp-10.1177_01461672251381184 – Supplemental material for I Get Knocked Down but I Get Up Again: Autonomous Motivation Sustains Identification and Collective Action After (Specific) Failure Supplemental material, sj-docx-1-psp-10.1177_01461672251381184 for I Get Knocked Down but I Get Up Again: Autonomous Motivation Sustains Identification and Collective Action After (Specific) Failure by Lisette Yip, Emma F. Thomas, Catherine Amiot, Léïla Eisner, Morgana Lizzio-Wilson, Winnifred R. Louis, Craig McGarty and Fathali Moghaddam in Personality and Social Psychology Bulletin

## Data Availability

The dataset for Study 1 is unavailable as participant consent was not obtained to publicly share the data. The datasets for Studies 2–3 are available in the Open Science Framework [https://osf.io/8ryk6/?view_only=c76508adba144666b96d34ceab7096c8].
