# Supplementary material for: I Get Knocked Down but I Get Up Again: Autonomous Motivation Sustains Identification and Collective Action After (Specific) Failure
Source: Pers Soc Psychol Bull. 2025 Nov 3;52(11):3581–97. doi: 10.1177/01461672251381184 (PMC13646426; doi:10.1177/01461672251381184)
Supplement: Supplementary file 1 [file sj-docx-1-psp-10.1177_01461672251381184.docx]

**Supplementary Materials**

**Section A: The Role of Introjected and External Regulation in Sustaining Identification and Collective Action After Failure**

We examined whether different types of controlled motivation would have distinct effects on opinion-based group identification and collective action, after failure. Although controlled motivation is considered as a broad category within self-determination theory, it can also be divided into two sub-types of regulation; external and introjected regulation. *External regulation* is the more externalised (less self-determined) of the two motives, and refers to behaviour that is driven purely by reward or punishment, while the behaviour or outcome itself holds no personal importance (Deci et al., 1999). *Introjected regulation* is sometimes considered to be partially internalised and is when the behaviour becomes relevant to one’s self-evaluation, e.g., eliciting feelings of shame, guilt, or pride, but is only valued because of its impact on these self-evaluations and is not fully internalised (Koestner et al., 1996). Previous research suggests that introjected and external regulations do not adequately reflect a single underlying construct of controlled motivation (Howard et al., 2017; Howard et al., 2020) and thus may have distinct effects on action (Yip et al., 2024). Thus, we also conducted exploratory analyses in all three studies to examine how these motives (i.e., external regulation and introjected regulation as two forms of controlled motivation) respectively influence identification and action, and how these effects may differ based on success and failure.

There were two items measuring introjected regulation (*r* = .46-.62) and two items measuring external regulation (*r* = .35-.60) in each study.

**Study 1**

We conducted two analyses using Model 8 in the PROCESS macro to test the interaction between introjected [external] regulation (IV) and condition (W) on opinion-based group identification (M) and collective action intentions (Y). There were no significant effects of introjected (Table 1) or external regulation (Table 2) on collective action intentions in any condition, and no interactions with outcome. There was a positive main effect of introjected regulation on opinion-based group identification; the indirect effect on collective action was not significant, *b* = 0.07, 95% *CI*[-0.01, 0.14], *SE* = 0.04.

**Table 1**

*Test of the Effects of Introjected Regulation on Collective Action Intentions via Opinion-Based Group Identification, Moderated by Outcome.*

|  | Opinion-based group identification T2 | |  | Collective action intentions T2 | |  |
| --- | --- | --- | --- | --- | --- | --- |
|  | *b* [95% *CI*] | *SE* | *p* | *b* [95% *CI*] | *SE* | *p* |
| Outcome (failure vs. success) | 0.09 [-0.51, 0.70] | 0.31 | .765 | -0.41 [-1.06, 0.24] | 0.33 | .212 |
| Opinion-based group identification T2 | - | - | - | **0.54 [0.39, 0.70]** | **0.08** | **< .001** |
| Introjected regulation T1 | **0.11 [0.003, 0.23]** | **0.06** | **.045** | 0.05 [-0.07, 0.17] | 0.06 | .443 |
| Outcome*Introjected regulation T1 | 0.00 [-0.11, 0.10] | 0.05 | .926 | 0.08 [-0.03, 0.19] | 0.06 | .153 |
| Opinion-based group identification T1 | **0.62 [0.48, 0.76]** | **0.07** | **< .001** | **-0.25 [-0.43, -0.07]** | **0.09** | **.006** |
| Collective action intentions T1 | 0.05 [-0.05, 0.15] | 0.05 | .323 | **0.78 [0.67, 0.89]** | **0.06** | **< .001** |

*Note*. Index of moderated moderated mediation -0.01 [-0.13, 0.12], *SE* = 0.06. Bold text denotes that confidence interval does not include zero.

**Table 2**

*Test of the Effects of External Regulation on Collective Action Intentions via Opinion-Based Group Identification, Moderated by Outcome.*

|  | Opinion-based group identification T2 | |  | Collective action intentions T2 | |  |
| --- | --- | --- | --- | --- | --- | --- |
|  | *b* [95% *CI*] | *SE* | *p* | *b* [95% *CI*] | *SE* | *p* |
| Outcome (failure vs. success) | 0.07 [-0.22, 0.36] | 0.15 | .620 | -0.05 [-0.36, 0.26] | 0.16 | .745 |
| Opinion-based group identification T2 | - | - | - | **0.54 [0.39, 0.70]** | **0.08** | **< .001** |
| External regulation T1 | 0.06 [-0.04, 0.15] | 0.05 | .239 | 0.06 [-0.04, 0.15] | 0.05 | .268 |
| Outcome*External regulation T1 | -0.00 [-0.09, 0.08] | 0.04 | .942 | 0.03 [-0.06, 0.13] | 0.05 | .497 |
| Opinion-based group identification T1 | **0.67 [0.54, 0.80]** | **0.07** | **< .001** | **-0.25 [-0.42, -0.07]** | **0.09** | **.006** |
| Collective action intentions T1 | 0.04 [-0.07, 0.14] | 0.05 | .458 | **0.76 [0.65, 0.87]** | **0.06** | **< .001** |

*Note*. Index of moderated moderated mediation = 0.00 [-0.12, 0.14], *SE* = 0.06. Bold text denotes that confidence interval does not include zero.

**Study 2**

We conducted two analyses using Model 8 identical to those in Study 1. There were no main effects of motivation or interactions between introjected regulation (Table 3) or external regulation (Table 4) and failure or success in any of these models.

**Table 3**

*Test of the Effects of Introjected Regulation on Collective Action Intentions via Opinion-Based Group Identification, Moderated by Failure and Success.*

|  | Opinion-based group identification T2 | | | Collective action intentions T2 | |  |
| --- | --- | --- | --- | --- | --- | --- |
|  | *b* [95% *CI*] | *SE* | *p* | *b* [95% *CI*] | *SE* | *p* |
| Failure (vs. control) | 0.05 [-0.49, 1.04] | 0.50 | .922 | 0.31 [-0.67, 1.30] | 0.50 | .532 |
| Success (vs. control) | -0.05 [-0.99, 0.89] | 0.47 | .916 | **-0.99 [-1.92, -0.06]** | **0.47** | **.037** |
| Opinion-based group identification T2 | - | - | - | **0.42 [0.25, 0.59]** | **0.09** | **< .001** |
| Introjected regulation T1 | 0.06 [-0.07, 0.20] | 0.07 | .338 | 0.08 [-0.05, 0.21] | 0.07 | .237 |
| Failure*Introjected regulation T1 | -0.03 [-0.20, 0.15] | 0.09 | .768 | -0.01 [-0.18, 0.16] | 0.09 | .897 |
| Success*Introjected regulation T1 | 0.01 [-0.15, 0.17] | 0.08 | .916 | 0.12 [-0.05, 0.28] | 0.08 | .157 |
| Opinion-based group identification T1 | **0.57 [0.42, 0.72]** | **0.07** | **< .001** | -0.10 [-0.28, 0.08] | 0.09 | .268 |
| Collective action intentions T1 | 0.10 [-0.03, 0.23] | 0.07 | .113 | **0.42 [0.29, 0.55]** | **0.07** | **< .001** |

*Note*. Index of moderated moderated mediation for failure = -0.01 [-0.09, 0.06], *SE* = 0.04. Index of moderated moderated mediation for success = 0.00 [-0.07, 0.08], *SE* = 0.04. Bold text denotes that confidence interval does not include zero.

**Table 4**

*Test of the Effects of External Regulation on Collective Action Intentions via Opinion-Based Group Identification, Moderated by Failure and Success.*

|  | Opinion-based group identification T2 | |  | Collective action intentions T2 | |  |
| --- | --- | --- | --- | --- | --- | --- |
|  | *b* [95% *CI*] | *SE* | *p* | *b* [95% *CI*] | *SE* | *p* |
| Failure (vs. control) | -0.27 [-0.73, 0.19] | 0.23 | .246 | 0.27 [-0.20, 0.73] | 0.23 | .254 |
| Success (vs. control) | 0.11 [-0.33, 0.56] | 0.23 | .614 | -0.26 [-0.71, 0.19] | 0.23 | .258 |
| Opinion-based group identification T2 | - | - | - | **0.43 [0.26, 0.61]** | **0.09** | **< .001** |
| External regulation T1 | 0.01 [-0.09, 0.11] | 0.05 | .786 | -0.09 [-0.19, 0.01] | 0.05 | .074 |
| Failure*External regulation T1 | 0.06 [-0.08, 0.20] | 0.07 | .388 | 0.00 [-0.13, 0.14] | 0.07 | .949 |
| Success*External regulation T1 | -0.04 [-0.18, 0.10] | 0.07 | .581 | -0.03 [-0.18, 0.11] | 0.07 | .629 |
| Opinion-based group identification T1 | **0.59 [0.46, 0.73]** | **0.07** | **< .001** | -0.04 [-0.22, 0.13] | 0.09 | .618 |
| Collective action intentions T1 | 0.10 [-0.03, 0.23] | 0.07 | .125 | **0.42 [0.29, 0.55]** | **0.07** | **< .001** |

*Note*. Index of moderated moderated mediation for failure = 0.03 [-0.04, 0.08], *SE* = 0.03. Index of moderated moderated mediation for success = -0.02 [-0.08, 0.06], *SE* = 0.04. Bold text denotes that confidence interval does not include zero.

**Study 3**

We conducted two analyses using Model 12 to test the interaction between introjected [external] regulation (IV), outcome (W) and framing (Z) on opinion-based group identification (M) and collective action intentions (Y). Autonomous motivation and external [introjected] regulation were entered as covariates, consistent with the tests of hypotheses in the manuscript.

We first tested the effects of introjected regulation. Effects are displayed in Table 5 and simple slopes in Table 6. There was a significant three-way interaction on collective action intentions, when controlling for opinion-based group identification. Tests of the simple interactions revealed that when framing was specific, the direct effect of introjected regulation on collective action was positive and greater for those in the success condition than those in the failure condition (*b* = -0.13, *F*(1, 366) = 8.72, *p* = .003) but there was no difference when framing was broad (*b* = 0.04, *F*(1, 366) = 0.57, *p* = .449). There were no interactions on opinion-based group identification, and no evidence of moderated moderated mediation.

**Table 5**

*Test of the Effects of Introjected Regulation on Collective Action Intentions via Opinion-Based Group Identification, Moderated by Outcome and Framing.*

|  | Opinion-based group identification | |  | Collective action intentions | |  |
| --- | --- | --- | --- | --- | --- | --- |
|  | *b* [95% *CI*] | *SE* | *p* | *b* [95% *CI*] | *SE* | *p* |
| Introjected regulation | **0.08 [0.002, 0.16]** | **0.04** | **.044** | **0.17 [0.09, 0.26]** | **0.04** | **< .001** |
| Outcome (failure vs. success) | -0.27 [-0.57, 0.04] | 0.15 | .085 | 0.30 [-0.03, 0.64] | 0.17 | .078 |
| Framing (specific vs. broad) | -0.04 [-0.35, 0.26] | 0.15 | .783 | -0.25 [-0.59, 0.09] | 0.17 | .152 |
| Opinion-based group identification | - | - | - | **0.63 [0.52, 0.75]** | **0.06** | **< .001** |
| Outcome*Introjected regulation | 0.05 [-0.01, 0.11] | 0.03 | .137 | -0.05 [-0.12, 0.02] | 0.03 | .166 |
| Framing*Introjected regulation | 0.00 [-0.06, 0.06] | 0.03 | .891 | 0.03 [-0.04, 0.10] | 0.03 | .387 |
| Outcome*Framing | -0.08 [-0.39, 0.22] | 0.15 | .584 | **0.49 [0.15, 0.83]** | **0.17** | **.005** |
| Outcome*Framing*Introjected regulation | 0.02 [-0.04, 0.08] | 0.03 | .568 | **-0.09 [-0.15, -0.02]** | **0.03** | **.012** |
| Autonomous motivation | **0.50 [0.41, 0.60]** | **0.05** | **< .001** | **0.14 [0.02, 0.26]** | **0.06** | **.024** |
| External regulation | 0.04 [-0.03, 0.10] | 0.04 | .307 | 0.06 [-0.01, 0.14] | 0.04 | .111 |

*Note*. Index of moderated moderated mediation = 0.01 [-0.04, 0.06], *SE* = 0.03. Index of moderated mediation (by outcome) in specific conditions = -0.04 [-0.02, 0.10], *SE* = 0.03. Index of moderated mediation (by outcome) in broad conditions = 0.02 [-0.07, 0.10], *SE* = 0.04. Bold text denotes that confidence interval does not include zero.

**Table 6**

*Simple Slopes Analysis of the Effects of Introjected Regulation by Condition.*

|  |  | Opinion-based group identification |  | Collective action intentions |  | Indirect effect |  |
| --- | --- | --- | --- | --- | --- | --- | --- |
|  |  | *b* [95% *CI*] | *SE* | *b* [95% *CI*] | *SE* | *b* [95% *CI*] | *SE* |
| Specific | Failure | **0.14 [0.02, 0.26]** | **0.06** | 0.07 [-0.07, 0.21] | 0.07 | **0.09 [0.004, 0.18]** | **0.05** |
|  | Success | 0.01 [-0.11, 0.13] | 0.06 | **0.34 [0.20, 0.47]** | **0.07** | 0.01 [-0.10, 0.12] | 0.06 |
| Broad | Failure | 0.11 [-0.02, 0.24] | 0.07 | **0.18 [0.04, 0.33]** | **0.07** | 0.07 [-0.06, 0.19] | 0.06 |
|  | Success | 0.06 [-0.09, 0.20] | 0.07 | 0.11 [-0.06, 0.27] | 0.08 | 0.04 [-0.06, 0.14] | 0.05 |

*Note*. Bold text denotes that confidence interval does not include zero.

We then tested the effects of external regulation. Effects are displayed in Table 7 and simple slopes in Table 8. There was a three-way interaction on opinion-based group identification; the simple interactions revealed that when framing was broad, there was a positive effect of external regulation on identification in the failure condition but not the success condition (*b* = 0.17, *F*(1, 367) = 11.84, *p* = .001). There was no difference in the specific framing conditions (*b* = -0.05, *F*(1, 367) = 1.40, *p* = .238). Additionally, the index of moderated moderated mediation was significant; there was a positive indirect effect of external regulation on collective action intentions, via opinion-based group identification, only in the broad-failure condition.

**Table 7**

*Test of the Effects of External Regulation on Collective Action Intentions via Opinion-Based Group Identification, Moderated by Outcome and Framing.*

|  | Opinion-based group identification | |  | Collective action intentions | |  |
| --- | --- | --- | --- | --- | --- | --- |
|  | *b* [95% *CI*] | *SE* | *p* | *b* [95% *CI*] | *SE* | *p* |
| External regulation | 0.04 [-0.03, 0.10] | 0.03 | .288 | 0.06 [-0.02, 0.14] | 0.04 | .118 |
| Outcome (failure vs. success) | **-0.20 [-0.39, -0.005]** | **0.10** | **.045** | **0.29 [0.07, 0.52]** | **0.11** | **.010** |
| Framing (specific vs. broad) | 0.13 [-0.06, 0.33] | 0.10 | .177 | -0.17 [-0.40, 0.05] | 0.11 | .133 |
| Opinion-based group identification | - | - | - | **0.63 [0.51, 0.74]** | **0.06** | **< .001** |
| Outcome*External regulation | 0.06 [-0.01, 0.12] | 0.03 | .095 | **-0.08 [-0.16, -0.01]** | **0.04** | **.033** |
| Framing*External regulation | **-0.08 [-0.14, -0.01]** | **0.03** | **.022** | 0.03 [-0.05, 0.10] | 0.04 | .458 |
| Outcome*Framing | **0.29 [0.10, 0.49]** | **0.10** | **.003** | 0.13 [-0.09, 0.36] | 0.12 | .249 |
| Outcome*Framing*External regulation | **-0.11 [-0.18, -0.04]** | **0.03** | **.001** | -0.02 [-0.10, 0.05] | 0.04 | .534 |
| Autonomous motivation | **0.50 [0.41, 0.60]** | **0.05** | **< .001** | **0.13 [0.002, 0.25]** | **0.06** | **.047** |
| Introjected regulation | **0.09 [0.01, 0.16]** | **0.04** | **.021** | **0.18 [0.10, 0.27]** | **0.04** | **< .001** |

*Note*. Index of moderated moderated mediation = -0.07 [-0.13, -0.01], *SE* = 0.03. Index of moderated mediation (by outcome) in specific conditions = -0.03 [-0.10, 0.02], *SE* = 0.03. Index of moderated mediation (by outcome) in broad conditions = 0.10 [0.01, 0.19], *SE* = 0.05. Bold text denotes that confidence interval does not include zero.

**Table 8**

*Simple Slopes Analysis of the Effects of External Regulation by Condition.*

|  |  | Opinion-based group identification |  | Collective action intentions |  | Indirect effect |  |
| --- | --- | --- | --- | --- | --- | --- | --- |
|  |  | *b* [95% *CI*] | *SE* | *b* [95% *CI*] | *SE* | *b* [95% *CI*] | *SE* |
| Specific | Failure | -0.10 [-0.22, 0.03] | 0.06 | -0.02 [-0.16, 0.13] | 0.07 | -0.06 [-0.14, 0.01] | 0.04 |
|  | Success | 0.01 [-0.12, 0.15] | 0.07 | **0.20 [0.05, 0.35]** | **0.08** | 0.01 [-0.07, 0.10] | 0.04 |
| Broad | Failure | **0.28 [0.15, 0.41]** | **0.07** | -0.02 [-0.18, 0.13] | 0.08 | **0.18 [0.02, 0.33]** | **0.08** |
|  | Success | -0.05 [-0.19, 0.09] | 0.07 | 0.09 [-0.07, 0.25] | 0.08 | -0.03 [-0.12, 0.05] | 0.04 |

*Note*. Bold text denotes that confidence interval does not include zero.

**Discussion**

In Studies 1 and 2, we found no effects of controlled forms of motivation on collective action intentions. However, in Study 3, introjected regulation positively predicted collective action intentions after experiencing specific success, though there was no evidence of mediation via identification. It may be that introjected motives are associated with feelings of pride after experiencing a clear success, which have been found to predict perceptions of group efficacy and subsequent collective action (Tausch & Becker, 2013). Previous research finds mixed effects of introjected regulation, with some studies reporting positive effects on behaviour and others reporting negative effects (see Howard et al., 2017 for a meta-analysis). It may be that its effects are more positive when the movement experiences success, but it does not sustain action over time (Yip et al., 2024) or through setbacks.

In Study 3, external regulation positively predicted identification and, in turn, collective action after failure, but only when failure was broad. External rewards which typically do not predict resilience in the face of setbacks (e.g., Holding et al., 2017; Leduc-Cummings et al., 2022) may play a role when a political movement is failing to reach its broader goals. It may be that external rewards such as social approval increase in importance as a motivator of collective action when the movement is failing because they are still attainable when the movement’s goals are not. However, it is worth noting that even in the broad-failure condition, the effect of external regulation on collective action intentions was weaker than that of autonomous motivation; and we found no effects of external regulation in Study 2. Thus, although we found some positive effects of controlled forms of motivation, the findings support the assertion that autonomous motivation is the strongest and most consistent driver of continued action in any context, and regardless of outcome.

Further research is needed to understand the role of controlled forms of motivation, which appear to have positive effects on opinion-based group identification and collective action when paired with autonomous motives (Yip et al., 2025), but may be detrimental to sustained action over time (Yip et al., 2024). The present studies do not suggest that controlled forms of motivation are detrimental to social movements, but provide further evidence that they are not effective in eliciting the same positive outcomes associated with autonomous motives.

**Section B: Supplementary Materials for Study 1**

**Table 9**

*Means and Correlations for Main Variables.*

|  | 1. | 2. | 3. | 4. | 5. | 6. | ‘No’ supporters  *M* (*SD*) | ‘Yes’ supporters  *M* (*SD*) |
| --- | --- | --- | --- | --- | --- | --- | --- | --- |
| 1. Autonomous motivation T1 | - |  |  |  |  |  | 6.05 (0.84) | 5.80 (1.00) |
| 2. Introjected regulation T1 | .68*** | - |  |  |  |  | 5.74 (1.14) | 5.62 (1.26) |
| 3. External regulation T1 | .05 | .15* | - |  |  |  | 3.04 (1.48) | 2.83 (1.31) |
| 4. Opinion-based group identification T1 | .57*** | .42*** | .20** | - |  |  | 5.52 (1.10) | 5.49 (1.02) |
| 5. Opinion-based group identification T2 | .51*** | .39*** | .21** | .67*** | - |  | 5.86 (1.21) | 5.72 (1.07) |
| 6. Collective action intentions T1 | .27*** | .21** | .27*** | .48*** | .36*** | - | 3.74 (1.42) | 4.03 (1.30) |
| 7. Collective action intentions T2 | .31*** | .25*** | .28*** | .43*** | .54*** | .75*** | 3.66 (1.62) | 3.71 (1.45) |

*Note*. * denotes significant correlation at *p* < .05, ** denotes *p* < .01, *** denotes *p* < .001.

**Section C: Supplementary Materials for Study 2**

**Table 10**

*Means and Correlations for Main Variables.*

|  | 1. | 2. | 3. | 4. | 5. | 6. | Failure  *M (SD)* | Success  *M (SD)* | Control  *M (SD)* |
| --- | --- | --- | --- | --- | --- | --- | --- | --- | --- |
| 1. Autonomous motivation T1 | - |  |  |  |  |  | 5.37 (1.00) | 5.74 (0.94) | 5.62 (1.01) |
| 2. Introjected regulation T1 | .51*** | - |  |  |  |  | 5.57 (1.30) | 5.60 (1.40) | 5.52 (1.17) |
| 3. External regulation T1 | -.06 | .12 | - |  |  |  | 3.11 (1.65) | 2.78 (1.53) | 2.71 (1.56) |
| 4. Opinion-based group identification T1 | .47*** | .35*** | .15* | - |  |  | 4.81 (1.06) | 5.15 (1.30) | 5.01 (1.04) |
| 5. Opinion-based group identification T2 | .50*** | .28*** | .08 | .63*** | - |  | 4.90 (1.15) | 5.21 (1.30) | 5.22 (0.95) |
| 6. Collective action intentions T1 | .30*** | .20** | .13 | .23** | .27*** | - | 3.90 (1.26) | 4.06 (1.32) | 4.40 (1.16) |
| 7. Collective action intentions T2 | .37*** | .27*** | -.03 | .26*** | .44*** | .53*** | 4.74 (1.03) | 4.33 (1.39) | 4.79 (1.05) |

*Note*. * denotes correlation is significant at *p* < .05, ** denotes *p* < .01, *** denotes *p* < .001.

**Analysis of the ‘Combined’ Condition Relative to ‘Control’ Condition**

We repeated a version of the test of hypotheses for Study 2 (Model 8) using a dichotomous independent variable testing the effects of membership in the combined condition (where participants were asked to reflect on both failure and success) with the control condition. First, the manipulation check did not show any differences in perceptions of success (*M* = 4.51, *SD* = 1.49, *p* = .647) or failure (*M* = 4.42, *SD* = 1.60, *p* = .986) between the combined condition and the control group. We tested whether the combined manipulation (W) predicted the effects of autonomous motivation (X) on action intentions (Y) via identification (M). There were no effects of combined (vs. control) condition or interactions with autonomous motivation on any dependent variables (Table 11). However, the simple slopes suggested that the effect of autonomous motivation on identification (*b* = 0.38 [0.09, 0.67], *SE* = 0.15, *p* = .012), and the indirect effect on action via identification (*b* = 0.10 [0.0001, 0.25], *SE* = 0.07) was positive and significant in the combined condition but not in the control condition.

**Table 11**

*Test of the Effects of Autonomous Motivation on Collective Action Intentions via Opinion-Based Group Identification, Moderated by Condition (Combined vs. Control).*

|  | Opinion-Based Group identification T2 | |  | Collective action intentions T2 | |  |
| --- | --- | --- | --- | --- | --- | --- |
|  | *b* [95% *CI*] | *SE* | *p* | *b* [95% *CI*] | *SE* | *p* |
| Combined (condition) | -0.58 [-1.65, 0.48] | 0.53 | .276 | -0.04 [-1.18, 1.09] | 0.57 | .940 |
| Opinion-based group identification T2 | - | - | - | **0.26 [0.02, 0.49]** | **0.12** | **.031** |
| Autonomous motivation T1 | **0.29 [0.07, 0.50]** | **0.11** | **.010** | 0.12 [-0.12, 0.36] | 0.12 | .326 |
| Combined*Autonomous motivation T1 | 0.09 [-0.10, 0.28] | 0.09 | .332 | -0.02 [-0.22, 0.18] | 0.10 | .837 |
| Opinion-based group identification T1 | **0.48 [0.28, 0.67]** | **0.10** | **< .001** | -0.04 [-0.27, 0.20] | 0.12 | .766 |
| Collective action intentions T1 | -0.02 [-0.18, 0.14] | 0.08 | .808 | **0.38 [0.21, 0.55]** | **0.09** | **< .001** |

*Note*. Index of moderated mediation = 0.05 [-0.05, 0.19], *SE* = 0.06. Bold text denotes that confidence interval does not include zero.

**Section D: Supplementary Materials for Study 3**

**Table 12**

*Cell Means and Correlation Matrix.*

|  | 1. | 2. | 3. | 4. | Specific  failure  *M (SD)* | Specific  success  *M (SD)* | Broad  failure  *M (SD)* | Broad  success  *M (SD)* |
| --- | --- | --- | --- | --- | --- | --- | --- | --- |
| 1. Autonomous motivation | - |  |  |  | 5.20 (1.24) | 5.21 (1.20) | 5.23 (1.20) | 5.21 (1.15) |
| 2. Introjected regulation | .62*** | - |  |  | 4.70 (1.55) | 4.80 (1.51) | 4.76 (1.50) | 4.89 (1.35) |
| 3. External regulation | -.17*** | .03 | - |  | 2.61 (1.34) | 2.55 (1.87) | 2.64 (1.35) | 2.84 (1.29) |
| 4. Identification | .61*** | .45*** | -.04 | - | 4.87 (1.19) | 4.98 (1.03) | 5.03 (1.15) | 5.12 (1.02) |
| 5. Action intentions | .53*** | .50*** | .03 | .66*** | 4.70 (1.22) | 4.47 (1.53) | 4.86 (1.40) | 4.94 (1.27) |

*Note*. * denotes correlation is significant at *p* < .05, ** denotes *p* < .01, *** denotes *p* < .001.

**Full Text for the Manipulation of Outcome and Framing**

*Specific-success condition*

**COP28 a success: countries commit to transitioning away from fossil fuels**

The 2023 United Nations Climate Change Conference (known as COP28) held in December last year was a success. For the first time ever, the final agreement between participating countries included acknowledgement of the need to transition away from fossil fuels in order to mitigate the worst effects of climate change. “Fossil fuels faced a reckoning at the U.N. climate negotiations,” said Ani Dasgupta, president of the World Resources Institute, in a statement. “This historic outcome marks the beginning of the end of the fossil fuel era.”

*Specific-failure condition*

**COP28 a failure: no commitment to phaseout of fossil fuels**

The 2023 United Nations Climate Change Conference (known as COP28) held in December last year was a failure. Despite the efforts of campaigners, the final agreement between participating countries failed to include any commitment to phase out fossil fuels in order to mitigate the worst effects of climate change. “Fossil fuels were the winner at the U.N. climate negotiations,” said Ani Dasgupta, president of the World Resources Institute, in a statement. “This disappointing outcome cements the continuation of the fossil fuel era.”

*Broad-success condition*

**Climate movement is succeeding: decades of campaigning are paying off**

The climate movement has enjoyed recent successes, achieving a range of milestones suggesting that its goals are in sight. Globally, investment in renewable energy continues to increase every year, and major falls in the cost of solar and wind energy suggest that the end of fossil fuels is near. For the first time ever, we have seen international consensus on the need to transition away from fossil fuels in order to mitigate the worst effects of climate change. President of the World Resources Institute, Ani Dasgupta, said in a statement that this is “the beginning of the end of the fossil fuel era,” suggesting that the climate movement is succeeding in achieving its goals.

*Broad-failure condition*

**Climate movement is failing: no progress after decades of campaigning**

The climate movement has experienced recent failures, reaching a range of milestones suggesting that it has failed to reach its key targets. Globally, carbon emissions continue to increase every year, and the approval of hundreds of new oil and gas projects suggests that the end of fossil fuels is nowhere in sight. Despite decades of effort from campaigners, there is still no international consensus on the need to phase out fossil fuels in order to mitigate the worst effects of climate change. President of the World Resources Institute, Ani Dasgupta, said in a statement that we are now “more likely than not” to pass the safe threshold of 1.5 degrees of global warming, suggesting that the climate movement is failing to achieve its goals.

**Exploratory Analysis of the Effects of Autonomous Motivation on Radical Action Intentions**

We conducted Model 12 in PROCESS to test the interaction between autonomous motivation (IV), outcome (W) and framing (Z) on identification (M) and radical action intentions (Y). Introjected regulation and external regulation were entered as covariates, consistent with the analyses of hypotheses for Study 3, and conventional collective action intentions were also entered as a covariate. There was no evidence of an indirect effect of autonomous motivation on radical action intentions, via identification (Table 13). While there was no overall direct effect, there were two-way interactions such that the effect of autonomous motivation on radical action intentions was stronger in the failure condition (vs. success) and in the broad condition (vs. specific). The simple slopes revealed a positive effect of autonomous motivation on radical action intentions only in the broad failure condition (Table 14).

**Table 13**

*Test of the Effects of Autonomous Motivation on Radical Action Intentions via Opinion-Based Group Identification, Moderated by Outcome and Framing.*

|  | Opinion-based group identification | |  | Radical action intentions | |  |
| --- | --- | --- | --- | --- | --- | --- |
|  | *b* [95% *CI*] | *SE* | *p* | *b* [95% *CI*] | *SE* | *p* |
| Autonomous motivation | **0.34 [0.25, 0.43]** | **0.05** | **< .001** | 0.03 [-0.12, 0.18] | 0.08 | .698 |
| Outcome (failure vs. success) | -0.23 [-0.57, 0.11] | 0.17 | .189 | **-0.68 [-1.22, -0.14]** | **0.27** | **.013** |
| Framing (specific vs. broad) | -0.25 [-0.59, 0.10] | 0.17 | .157 | **0.60 [0.06, 1.14]** | **0.27** | **.029** |
| Opinion-based group identification | - | - | - | 0.00 [-0.16, 0.16] | 0.08 | .977 |
| Outcome*Autonomous motivation | 0.03 [-0.03, 0.10] | 0.03 | .335 | **0.13 [0.03, 0.23]** | **0.05** | **.010** |
| Framing*Autonomous motivation | 0.05 [-0.02, 0.11] | 0.03 | .165 | **-0.11 [-0.21, -0.01]** | **0.05** | **.039** |
| Outcome*Framing | **-0.47 [-0.81, -0.13]** | **0.17** | **.007** | 0.34 [-0.20, 0.88] | 0.28 | .214 |
| Outcome*Framing*Autonomous motivation | **0.08 [0.02, 0.15]** | **0.03** | **.010** | -0.07 [-0.17, 0.03] | 0.05 | .153 |
| Introjected regulation | -0.01 [-0.08, 0.05] | 0.04 | .683 | **-0.17 [-0.28, -0.07]** | **0.05** | **.002** |
| External regulation | 0.00 [-0.06, 0.06] | 0.03 | .876 | **0.42 [0.32, 0.51]** | **0.05** | **< .001** |
| Collective action intentions | **0.38 [0.31, 0.45]** | **0.04** | **< .001** | **0.47 [0.35, 0.59]** | **0.06** | **< .001** |

*Note*. Index of moderated moderated mediation = 0.00 [-0.01, 0.01], *SE* = 0.01. Index of moderated mediation (by outcome) in specific conditions = -0.00 [-0.02, 0.02], *SE* = 0.01. Index of moderated mediation (by outcome) in broad conditions = 0.00 [-0.01, 0.01], *SE* = 0.01. Bold text denotes that confidence interval does not include zero.

**Table 14**

*Simple Slopes Analysis of the Effects of Autonomous Motivation by Condition*.

|  |  | Opinion-based group identification | | Radical action intentions | | Indirect effect | |
| --- | --- | --- | --- | --- | --- | --- | --- |
|  |  | *b* [95% *CI*] | *SE* | *b* [95% *CI*] | *SE* | *b* [95% *CI*] | *SE* |
| Specific | Failure | **0.50 [0.37, 0.64]** | **0.07** | -0.02 [-0.24, 0.21] | 0.12 | 0.00 [-0.07, 0.07] | 0.04 |
|  | Success | **0.27 [0.13, 0.41]** | **0.07** | -0.14 [-0.35, 0.08] | 0.11 | 0.00 [-0.04, 0.04] | 0.02 |
| Broad | Failure | **0.24 [0.09, 0.39]** | **0.07** | **0.34 [0.11, 0.57]** | **0.12** | 0.00 [-0.04, 0.05] | 0.02 |
|  | Success | **0.35 [0.19, 0.50]** | **0.35** | -0.07 [-0.32, 0.17] | 0.12 | 0.00 [-0.06, 0.05] | 0.03 |

*Note*. Bold text denotes that confidence interval does not include zero.

**References**

Deci, E. L., Koestner, R., & Ryan, R. M. (1999). A meta-analytic review of experiments examining the effects of extrinsic rewards on intrinsic motivation. *Psychological Bulletin, 125*(6), 627. <https://doi.org/10.1037/0033-2909.125.6.627>

Holding, A. C., Hope, N. H., Harvey, B., Marion Jetten, A. S., & Koestner, R. (2017). Stuck in limbo: Motivational antecedents and consequences of experiencing action crises in personal goal pursuit. *Journal of Personality, 85*(6), 893-905. <https://doi.org/10.1111/jopy.12296>

Howard, J. L., Gagné, M., & Bureau, J. S. (2017). Testing a continuum structure of self-determined motivation: A meta-analysis. *Psychological Bulletin, 143*(12), 1346. <https://doi.org/10.1037/bul0000125>

Howard, J. L., Gagné, M., Van den Broeck, A., Guay, F., Chatzisarantis, N., Ntoumanis, N., & Pelletier, L. G. (2020). A review and empirical comparison of motivation scoring methods: An application to self-determination theory. *Motivation and Emotion, 44*(4), 534-548. <https://doi.org/10.1007/s11031-020-09831-9>

Koestner, R., Losier, G. F., Vallerand, R. J., & Carducci, D. (1996). Identified and introjected forms of political internalization: extending self-determination theory. *Journal of Personality and Social Psychology, 70*(5), 1025-1036. <https://doi.org/10.1037/0022-3514.70.5.1025>

Leduc-Cummings, I., Werner, K. M., Milyavskaya, M., Dominick, J. K., & Cole, S. (2022). Experiencing obstacles during goal pursuit: The role of goal motivation and trait self-control. *Journal of Research in Personality, 99*, 104231. <https://doi.org/10.1016/j.jrp.2022.104231>

Tausch, N., & Becker, J. C. (2013). Emotional reactions to success and failure of collective action as predictors of future action intentions: A longitudinal investigation in the context of student protests in Germany. *British Journal of Social Psychology, 52*(3), 525-542. <https://doi.org/10.1111/j.2044-8309.2012.02109.x>

Yip, L., Thomas, E. F., Amiot, C., Louis, W. R., & McGarty, C. (2024). Autonomous motives foster sustained commitment to action: Integrating self-determination theory and the social identity approach. *Personality and Social Psychology Bulletin, 50*(5), 750-765. <https://doi.org/10.1177/01461672221148396>

Yip, L., Thomas, E. F., Bliuc, A. M., Boza, M., Kende, A., Lizzio‐Wilson, M., Reese, G., & Smith, L. G. (2025). Motivations to engage in collective action: A latent profile analysis of refugee supporters. *British Journal of Social Psychology, 64*(1), e12786. <https://doi.org/bjso.12786>
